# Antitumor Macrophage Response to *Bacillus pumilus* Ribonuclease (Binase)

**DOI:** 10.1155/2017/4029641

**Published:** 2017-05-18

**Authors:** Anna Makeeva, Julian Rodriguez-Montesinos, Pavel Zelenikhin, Alexander Nesmelov, Klaus T. Preissner, Hector A. Cabrera-Fuentes, Olga N. Ilinskaya

**Affiliations:** ^1^Department of Microbiology, Kazan Federal University, Kremlevskaya Str. 18, Kazan 420008, Russia; ^2^Institute of Biochemistry, Medical School, Justus-Liebig-University, Friedrichstrasse 24, 35390 Giessen, Germany; ^3^Cardiovascular and Metabolic Disorders Program, Duke-National University of Singapore, 8 College Road, Singapore 169857; ^4^Escuela de Ingenieria y Ciencias, Centro de Biotecnologia-FEMSA, Tecnologico de Monterrey, Monterrey, NL, Mexico

## Abstract

Extracellular bacterial ribonucleases such as binase from *Bacillus pumilus* possess cytotoxic activity against tumor cells with a potential for clinical application. Moreover, they may induce activation of tumor-derived macrophages either into the M1-phenotype with well-documented functions in the regulation of the antitumor immune response or into M2-macrophages that may stimulate tumor growth, metastasis, and angiogenesis. In this study, binase or endogenous RNase1 (but not RNA or short oligonucleotides) stimulated the expression of activated NF-*κ*B p65 subunit in macrophages. Since no changes in MyD88 and TRIF adaptor protein expression were observed, toll-like receptors may not be involved in RNase-related NF-*κ*B pathway activation. In addition, short exposure (0.5 hr) to binase induced the release of cytokines such as IL-6, МСР-1, or TNF-*α* (but not IL-4 and IL-10), indicative for the polarization into antitumor M1-macrophages. Thus, we revealed increased expression of activated NF-*κ*B p65 subunit in macrophages upon stimulation by binase and RNase1, but not RNA or short oligonucleotides.

## 1. Introduction

Macrophages constitute a large proportion of the immune cell infiltrate which is present in many tumors [1, 2]. During the course of malignancy progression, macrophages migrate towards the site of tumor development and promote the immune response activation against neoplasm. The cytotoxicity of macrophages toward tumor cells requires stimulation either with products of bacterial cell wall or with endogenous cytokines. Activated macrophages secrete a number of substances that participate directly in tumor cell killing [3, 4]. However, macrophages, associated with tumors, may induce tumor cell proliferation by direct growth factor secretion or they may affect endothelial cells to provoke tumor tissue neovascularization [5]. Chemotherapy and radiotherapy can have dual influences on tumor-associated macrophages, in that, a misdirected macrophage-orchestrated tissue repair response can result in chemoresistance, but under other circumstances, tumor-associated macrophages are essential for effective therapy [6].

Xenogeneic, extracellular cytotoxic ribonucleases (RNases) of bacterial or eukaryotic origin, that trigger apoptosis/cytotoxicity in tumor and other cells, may have a great potential as anticancer drugs [7–11]. We earlier demonstrated the selective cytotoxic action of the RNase binase from *Bacillus pumilus* (formerly designated *B. intermedius*) towards lung carcinoma and leukemic and ovarian cancer cells [8, 12–14] and found that cell sensitivity towards xenogeneic RNases is related to expression of oncogenes ras, kit, and AML1 [15–17].

In murine peripheral blood monocytes/macrophages, binase induced synthesis of proinflammatory cytokines including monocyte chemoattractant protein-1 (MCP-1), tumor necrosis factor-*α* (TNF-*α*), and interleukin-6 (IL6), but not anti-inflammatory IL10 [18]. Cellular survival is associated with NF-*κ*B-related signal transduction [19], whereas binase-induced apoptosis of leukemic Kasumi cells was accomplished by enhanced expression of TNF-*α* (as an agonist of NF-*κ*B signaling), NF*κ*B2, and RELB [14]. The latter two proteins form a heterodimeric complex and contribute to the activation of noncanonical NF-*κ*B signaling [20]. At the same time, binase promoted increased expression of inhibitors of this pathway, including NF*κ*BIA (I*κ*-B protein which inactivates NF-*κ*B by trapping it in the cytoplasm) as well as NF*κ*BIZ and BCL3 [21], indicative for the activation of counteracting pathways in cells that would lead to signal commotion with the result of cell death.

Based on these somehow paradoxical observations, binase-related activation of NF-*κ*B in human and mouse macrophage model cell lines THP-1 and RAW264.7 was investigated to reveal a role of this process in antitumor effects of binase.

## 2. Methods

### 2.1. Enzymes

Binase (monomeric form of 12.3 kDa, 109 amino acid residues, pI value of 9.5) was isolated as homogenous protein from cultural fluid, according to the procedure described by Makarov et al. [22], and enzyme purity was confirmed by electrophoresis [7, 8, 12, 13]. Catalytic activity of binase toward yeast RNA was 14 × 106 U/mg protein [23]. Bovine pancreas RNase1 was purchased from Thermo Scientific (сatalytic activity toward yeast RNA was ≥5000 U/mg protein).

### 2.2. Cell Cultures

THP-1, a human monocytic cell line derived from an acute monocytic leukemia patient, and RAW 264.7 cells (Abelson murine leukemia virus-transformed macrophages) were obtained from American Type Culture Collection (Rockville, MD). THP-1 cells were maintained in RPMI 1640 and supplemented with 10% fetal bovine serum (FBS), 1% penicillin-streptomycin, and 1% L-glutamine (Invitrogen) at 37°C in 5% CO_2_. For experiments, cells were seeded at a density of 1 × 10^4^ cells/well (96-well plates) or 5 × 10^5^ cells/well (12-well plates) in an antibiotic-free medium and differentiated into macrophages using phorbol 12-myristate 13-acetate (PMA, Sigma) at 2 *μ*g/ml for 48 h. RAW 264.7 murine macrophages were maintained in Dulbecco's modified Eagle medium, high glucose, L-glutamine, and NaPyr (Invitrogen) supplemented with 10% heat-inactivated FBS and were seeded into 96-well plates at density of 1 × 104 cells/well or into 12-well plates at a density of 1 × 105 cells/well. RAW 264.7 macrophages were activated with 30 U/mL interferon-*γ* (Calbiochem) and 10 ng/mL lipopolysaccharide (LPS; Sigma).

### 2.3. Cell Viability

The viability of macrophages was determined using the Cytotoxicity Detection Kit (Roche) based on the measurement of lactate dehydrogenase (LDH) released from damaged cells and the XTT cell proliferation assay (Life Technologies) based on a conversion of the tetrazolium dye XTT to reduce colored form by transplasma membrane electron transport system. All procedures were performed in accordance with standard protocols. Briefly, cells were seeded into 96-well plates, allowed to reach 60% confluence, and treated with chemicals dissolved in fresh FBS-free medium as indicated. For XTT-analysis, 25 *μ*l of XTT solution were added to each well and incubated for 4 h at 37°C. To determine the LDH activity, 100 *μ*l of cultural medium after cell sedimentation was incubated with 100 *μ*l of LDH solution for 25 min at room temperature in the dark. For both assays, absorbance at 450/630 nm was measured with ELx80 Absorbance Microplate Reader (BioTek). Cell death was expressed as the percentage of LDH release, which was calculated using the following formula: percentage of LDH release = 100 × (test LDH release − spontaneous release)/(maximum release − spontaneous release) as previously described [8]. The maximum release was determined by dissolution of cell monolayers using 1% (vol/vol) Triton X-100. For XTT assay, the viability was expressed as a percentage of control cell values.

### 2.4. RNA Extraction, Hydrolysis, and Capillary Electrophoresis

Total RNA was extracted from A549 human adenocarcinoma cells using peqGOLD Total RNA kit. All the experiments with RNA were performed in RNase-free water. Quality of extracted RNA was checked using Nanodrop 2000 (Thermo Scientific) and capillary electrophoresis. Starting RNA concentration before hydrolysis was 500 *μ*g/ml, RNase1 and binase were added at indicated concentrations, and the samples were incubated at 37°C, 300 rpm for 60 min. The reaction products were stored at 0°C when catalytic activity of RNases is reduced and immediately loaded onto the chip for capillary electrophoresis. Alternatively, samples were immediately added into cell medium for treatment. Capillary electrophoresis was performed using Agilent 2100 Bioanalyzer and Agilent RNA 6000 Nano Kit.

### 2.5. Western Blot Analysis

Samples were obtained using RIPA buffer supplemented with complete proteinase cocktail (Roche), 0.5 mM phenylmethanesulfonyl fluoride (PMSF), and 2 mM sodium orthovanadate. Extracts were sonicated and stored at −80°C until use. Protein contents were determined by the Bradford method [24] using bovine serum albumin as standard. Twenty *μ*g per lane of total protein was submitted to 12% SDS–polyacrylamide gel electrophoresis and subsequently blotted onto polyvinylidene difluoride membranes. Membranes were blocked with TBS containing 5% skim milk or 5% BSA, 50 mM Tris HCl, 150 mM NaCl, 0.1% Tween 20, pH 7.6 for 1 h at room temperature. Immunoblotting was performed using rabbit anti-MyD88 (1 : 1000), anti-NF-*κ*B (p65, 1 : 1000), anti-phospho-NF-*κ*B (p65 subunit, phosphorylated at Ser536, 1 : 1000), anti-NF-*κ*B2 (p100/p52, 1 : 1000), anti-TRIF (1 : 1000), and rat anti-*β*-actin (1 : 1000) antibodies. All antibodies were purchased from Cell Signalling. Appropriate secondary antibodies (1 : 5000) conjugated with horse radish peroxidase (Sigma) and ECL reagent (Amersham) were used for detection. All procedures were performed in accordance with standard manufacturer protocols. The ImageLab software was used for quantification of proteins; background was subtracted using rolling disc algorithm. Expression of all analyzed proteins was normalized to *β*-actin level.

### 2.6. Statistics

Data were analyzed by unpaired one-way analysis of variance (ANOVA) followed by Tukey's, Dunnett's, or Bonferroni's multiple comparisons test, when appropriate, to determine statistical significance of the differences using GraphPad Prism, GraphPad Software (La Jolla, CA, USA) (www.graphpad.com). Differences with *p* < 0.05 were considered to be statistically significant.

## 3. Results

### 3.1. Exogenous RNase1, Binase, and RNA Do Not Affect Macrophage Survival

The influence of exogenous RNases (binase and RNase1), extracellular RNA, and products of RNA hydrolysis (short oligonucleotides) on growth and viability of THP-1 and RAW234.7 macrophages was examined using the XTT reduction assay and LDH release test. These assays are sensitive enough to be applied to cells grown in 96-well plates and correlate well with cell number (data not shown). Treatment of macrophages with total RNA from A549 cells at 10 *μ*g/ml, RNase1, binase or short RNA fragments obtained after RNA hydrolysis by RNase1, and binase did not change the viability of RAW264.7 or THP-1 cells after 3 h or 6 h, whereas 1 *μ*M staurosporine (positive control) induced significant cell death (about 60%) after 6 h exposure. When exposed to exogenous RNase1 (0.01–10 *μ*g/ml), binase (0.01–100 *μ*g/ml), or RNA (0.01–10 *μ*g/ml) and oligonucleotides, no LDH leakage was observed (data not shown). Thus, exogenous RNase1, binase, and total RNA extracted from A549 cells were not harmful towards THP-1 and RAW264.7 macrophages.

### 3.2. Binase and RNase1 Induce Different RNA Digestion Profiles

**Figure 1:**
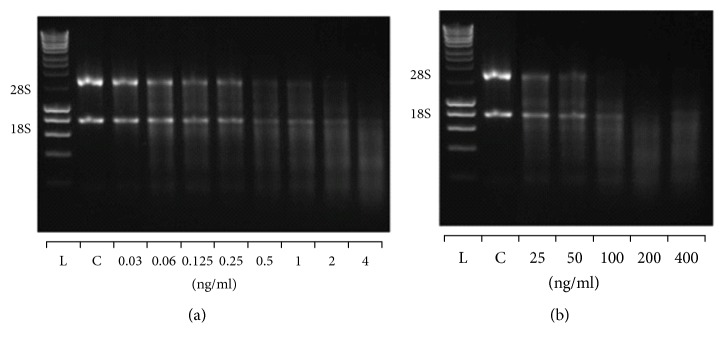
Agarose gel electrophoresis of RNA fragments patterns obtained by catalytic action of RNase1 (a) or binase (b) at indicated concentrations. Reaction was performed in RNase-free water and incubated for 10 min at 37°C with shaker at low speed (300 rpm). Starting RNA concentration before hydrolysis was 500 *μ*g/ml (control, C). L: RNA HyperLadder 1 kb (Bioline). Representative image of three experiments is shown.

**Figure 2:**
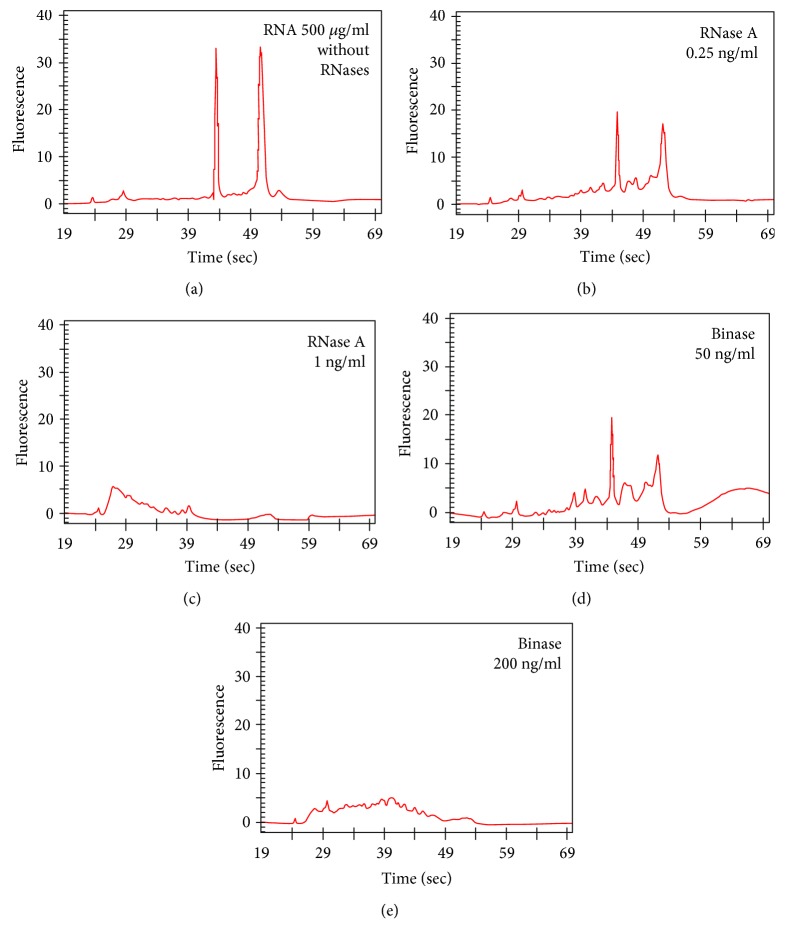
Capillary electrophoresis of RNA fragment patterns obtained by catalytic action of RNase1 (b), (c) or binase (d), (e) at indicated concentrations. Starting RNA concentration before hydrolysis was 500 *μ*g/ml (a). Reaction was performed in RNase-free water and incubated for 10 min at 37°C with shaker at low speed (300 rpm).

Since products of catalytic RNA digestion by RNase1 or binase may be relevant as to their effect on macrophages, hydrolysis of total RNA (from A549 cells) by binase and RNase1 within a wide concentration range of both RNases was performed. Upon electrophoretic analysis, in the absence of RNases (control), total RNA revealed two major bands, corresponding to the 28S and 18S ribosomal RNA (Figures 1(a) and 1(b)). The 28S RNA-band disappeared during the 1 h hydrolysis either at 2 ng/ml RNase1 (Figure 1(a)) or at 100 ng/ml binase (Figure 1(b)), indicating that RNase1 has a higher specific catalytic activity compared to binase. At low concentrations of both RNases, similar RNA fragmentation profiles appeared (Figures 2(b) and 2(d)), while at higher enzyme concentrations, the profiles of generated low-molecular weight oligonucleotides differed considerably (Figures 2(c) and 2(e)). Still, it remains unknown whether such differences in the profiles of RNA fragments generated by RNase1 and binase may contribute to their different biological effects, since noncytotoxic pancreatic RNases demonstrated very low immunogenicity [25], did not bind to the cell surface, and were not internalized into cell [26], whereas cytotoxic binase bound to cell membrane receptors, for example, RAS protein subfamily receptors, and effectively permeated cells [8, 27].

### 3.3. RNases but Not the Cleaved or Native RNA Activate NF-*κ*B System in Macrophages

Based on previous data from our group on noncanonical NF-*κ*B signaling pathway in leukemic cells [17], we propose that binase and RNase1 and/or RNA hydrolysis products exhibit cellular activity, including promotion of NF-*κ*B signaling in macrophages.

**Figure 3:**
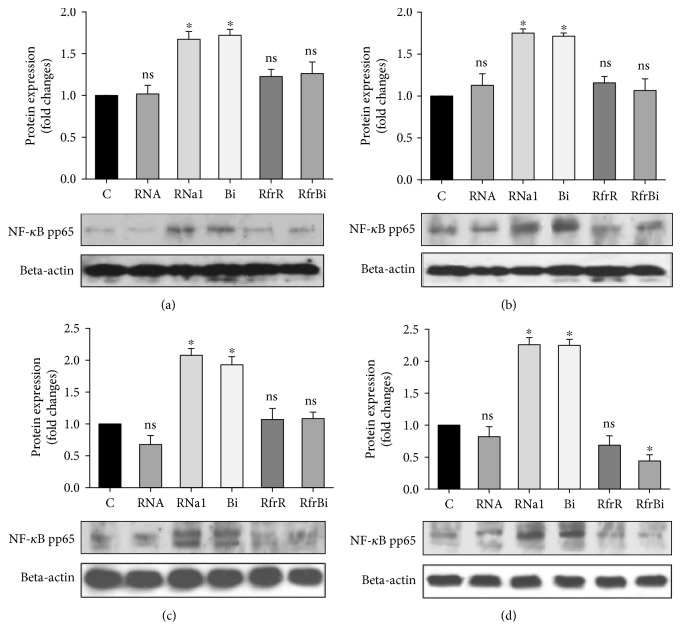
Quantification of phospho-p65 NF-*κ*B protein (pp65) level in RAW 264.7 macrophages (a), (b) and THP-1-derived macrophages (c), (d) after 0.5 h (a), (c) or 3 h (b), (d) of treatment. Macrophages were treated for indicated time with RNAase1, binase, total RNA from A549 adenocarcinoma cells (10 *μ*g/ml), and with RNA fragments obtained after RNA (500 *μ*g/ml) hydrolysis by binase (2 ng/ml) or RNase1 (100 ng/ml) at 37°C, 300 rpm for 60 min. After, treatment cells were harvested and the protein level was measured using the Western blotting. Representative image of three independent experiments are shown. С: control (untreated cells); RNa1: RNase1 (10 *μ*g/ml); Bi: binase (10 *μ*g/ml); RfrR: RNA fragments, obtained after RNA hydrolysis by RNase1; RfrBi: RNA fragments, obtained after RNA hydrolysis by binase. Data represent mean ± SD (*n* = 3), ^∗^*p* < 0.05, ns = nonsignificant.

Cells were stimulated with intact or hydrolyzed RNA, with RNase1 or binase alone in each case for 0.5 h, 1 h, or 3 h, and the phosphorylation of NF-*κ*B pathway-related proteins was quantified by Western blotting. When stimulated with exogenous RNases, the level of phosphorylated serine-536 on p65-subunit of NF-*κ*B (pp65) was increased (1.7 fold) in RAW264.7 (Figures 3(a) and 3(b)) and by 2.3 fold in THP-1 cells (Figures 3(c) and 3(d)); pp65 levels were apparent after 0.5 h and remained high for 3 h of stimulation. The expression of pp65 was not elevated when macrophages were stimulated by total extracellular RNA or hydrolysis generated by both RNases (Figure 3).

**Figure 4:**
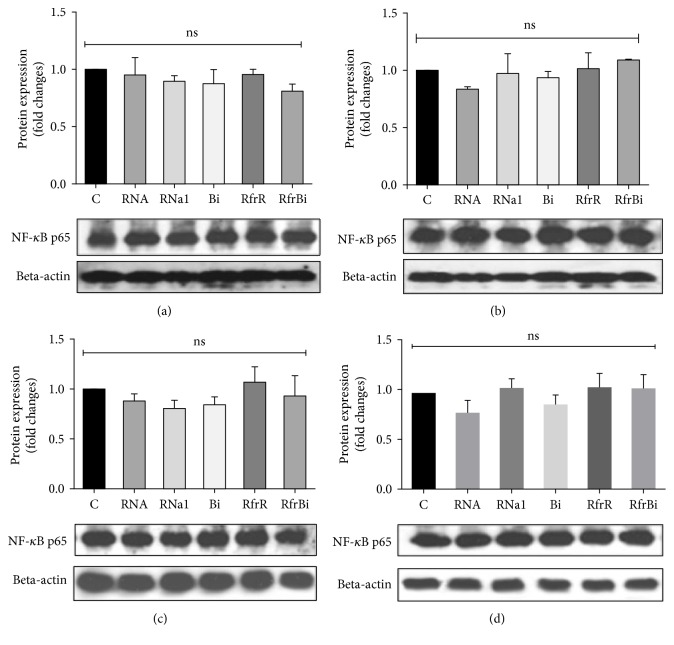
Quantification of p65 NF-*κ*B protein level in RAW 264.7 macrophages (a), (b), THP-1-derived macrophages (c), (d) after 0.5 h (a), (c) or 3 h (b), (d) of treatment. Macrophages were treated for indicated time with RNase1, binase, total RNA from A549 adenocarcinoma cells (10 *μ*g/ml), and with RNA fragments obtained after RNA (500 *μ*g/ml) hydrolysis by binase (100 ng/ml) or RNase1 (2 ng/ml) at 37°C, 300 rpm for 60 min. After, treatment cells were harvested and the protein level was measured using the Western blotting. Representative image of three independent experiments are shown. С: control (untreated cells); RNa1: RNase1 (10 *μ*g/ml); Bi: binase (10 *μ*g/ml); RfrR: RNA fragments, obtained after RNA hydrolysis by RNase1; RfrBi: RNA fragments, obtained after RNA hydrolysis by binase. Data represent mean ± SD (*n* = 3), ns = nonsignificant.

In control experiments, there were no significant changes observed in the level of nonphosphorylated NF-*κ*B p65-subunit in macrophages when stimulated with binase, RNase1, or intact extracellular RNA and RNA fragments as compared to untreated cells (Figures 4(a)–4(d)). Thus, binase and RNase1 but not the products of their catalyzed RNA hydrolysis induced the level of activated NF-*κ*B p65-subunit.

**Figure 5:**
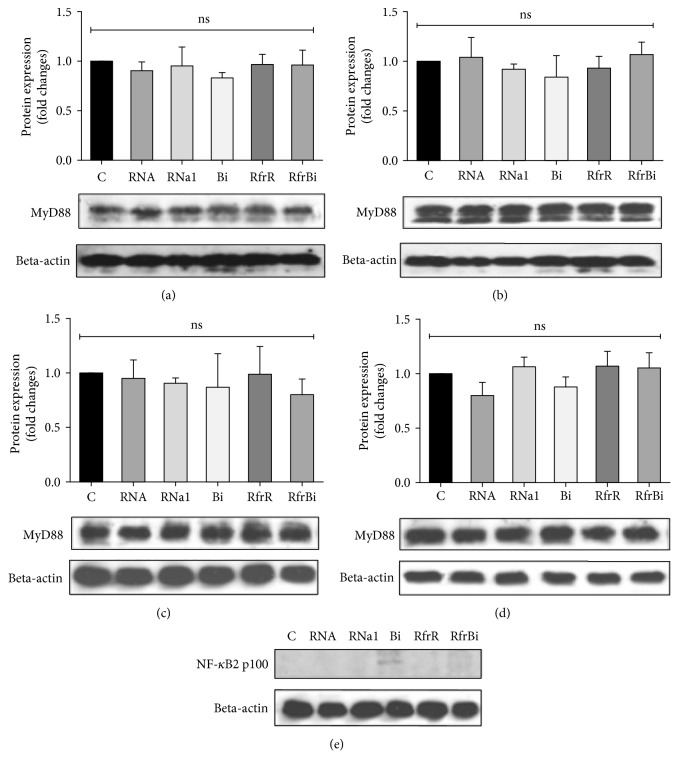
Quantification of MyD88 protein level in RAW 264.7 macrophages (a), (b), THP-1-derived macrophages (c), (d) after 0.5 h (a), (c) or 3 h (b), (d) of treatment and p100/p52 2NF-*κ*B protein level in THP-1 macrophages after 0.5 h of treatment (e). Macrophages were treated for indicated time with RNase1, binase, total RNA from A549 adenocarcinoma cells, and with RNA fragments obtained after RNA (500 *μ*g/ml) hydrolysis by binase (100 ng/ml) or RNase1 (2 ng/ml) at 37°C, 300 rpm for 60 min. After, treatment cells were harvested and the protein level was measured using the Western blotting. Representative image of three independent experiments are shown. С: control (untreated cells); RNa1: RNase1 (10 *μ*g/ml); Bi: binase (10 *μ*g/ml); RfrR: RNA fragments, obtained after RNA hydrolysis by RNase1; RfrBi: RNA fragments, obtained after RNA hydrolysis by binase. Data represent mean ± SD (*n* = 3), ns = nonsignificant.

To investigate whether RNases, total RNA, or RNA hydrolysis fragments are involved in noncanonical NF-*κ*B signaling by regulating the processing of p100 to p52, THP-1 macrophages were treated with the indicated agonist and Western blots were probed for p100/p52. While treatment with binase resulted in low level of p100 protein, no p52 protein was detectable (Figure 5(e)). At later time points, expression of p100 protein decreased to undetectable levels (data not shown). Thus, none of the agents under consideration stimulated the processing of p100 to p52.

We did not find any correlation between myeloid differentiation factor 88 (MyD88) adapter protein level, which is participating in signaling from toll-like receptors (TLRS) except TLR 3, in nontreated macrophages and macrophages treated with RNase 1, binase, or RNA/oligonucleotides (Figures 5(a)–5(d)). Next, we assessed TIR-domain-containing adapter-inducing interferon-*β* (TRIF) functioning downstream of both TLR3 and TLR4. Neither RNA and oligonucleotides nor RNase 1 and binase induced increased expression of TRIF in macrophages (data not shown).

### 3.4. Binase Promotes the Release of IL-6, TNF-*α*, and MCP-1 in THP-1-Derived Macrophages

**Figure 6:**
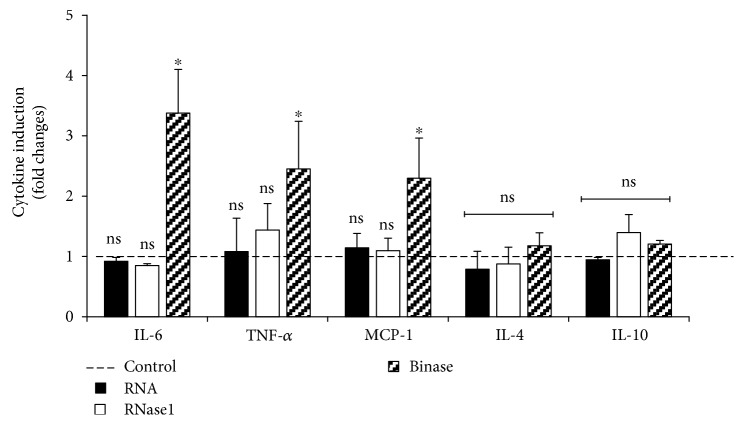
Binase-induced release of proinflammatory mediators in human THP-1-derived macrophages. THP-1-derived macrophages were treated in the absence (control) or presence of RNA (10 *μ*g/ml), RNase1 (10 *μ*g/ml), or binase (10 *μ*g/ml) during 0.5 h, followed by a supernatant proteome/cytokine profiler array (R&D Systems). Values represent mean ± SD of the mean from three independent experiments performed in triplicates. ^∗^*p* < 0.05 versus control, ns = nonsignificant.

To delineate these results, a significant increase in the release of proinflammatory mediators after short-term treatment (0.5 h) with binase (10 *μ*g/ml) in a cell culture model of THP-1-derived macrophages was observed (Figure 6). Exposure towards binase enhanced the release of IL-6, TNF-*α*, MCP-1, but not IL-4 and IL-10. RNA of RNase 1 did not influence basal levels of the pro- and anti-inflammatory mediators.

### 3.5. Binase-Induced Macrophage M1-Polarization

**Figure 7:**
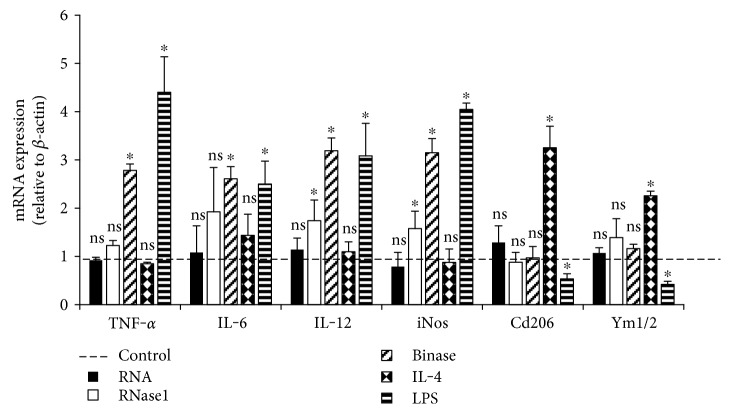
Influence of binase treatment on macrophage polarization markers and proinflammatory mediators in THP-1-derived macrophages. THP-1 monocytes were differentiated in the presence of PMA-conditioned medium and exposed to medium only (control, dotted line) or to RNA (10 μg/ml), RNase1 (10 *μ*g/ml), binase (10 *μ*g/ml), LPS (100 ng/ml), or IL-4 (10 ng/ml) for 0.5 h as indicated. In all cases, mRNA expression of TNF-α, IL-6, IL-12, iNos, Cd206, and Ym1/2 was analyzed by real-time PCR. Data are expressed as changes in the ratio between target gene and *β-actin* mRNA expression. The results were obtained from six independent experiments carried out in duplicates. Values represent mean ± SD. ^∗^*p* < 0.05 versus control, ns = nonsignificant.

Upon exposure to binase, PMA-mediated differentiation of THP-1 monocytes was switched to the M1-phenotype, resulting in inflammatory markers *TNF-α*, *Il-6*, and *Il-12* and inducible nitric oxide synthase (*iNos*) upregulation (Figure 7()). Ten *μ*g/ml binase as well as LPS induced M1-polarization and upregulation of proinflammatory genes to the same extent. In comparison, exposure of M2-polarized THP-1 towards the anti-inflammatory cytokine IL-4 induced typical M2-markers such as macrophage mannose receptor-2 CD206 and chitinase-like proteins Ym1/2 (Figure 7()). Together, these findings underline the nature of binase as a proinflammatory agonist, while its exact mode of action that engages several signaling pathways remains to be clarified.

## 4. Discussion

Cytotoxic exogenous RNase binase triggering apoptotic response in malignant cells has potential as anticancer drug. Among all the studied cells, cells of acute myeloid leukemia expressing the KIT oncogene were the most sensitive to the toxic actions of binase; Kasumi-1 cells undergo apoptosis induced by binase at concentration 0.5 *μ*g/ml [14–16]. In Kasumi-1 cells, binase switched on a number of regulatory mechanisms including noncanonical NF-*κ*B signaling pathway [17]; in T-cell acute lymphoblastic leukemia Jurkat, binase induced apoptosis and reduced transcription factor NF-*κ*B level [28]; therefore, NF-*κ*B signaling apparently is a common feature of the action of RNases on cancer cells.

Here, we analyze the role of binase in macrophage activation through canonical NF-*κ*B pathway using detection of pp65 subunit forming heterodimeric complexes with NFKB1, the most abundant form of NF-*κ*B. First of all, we demonstrated that binase at concentration sufficient for cytotoxic action toward leukemic cells did not inhibit macrophages viability and did not destroy their integrity measured by extracellular release of lactate dehydrogenase. Treatment of macrophages with extracellular RNA and exogenous RNase1 did not affect macrophages viability as well.

Macrophages are the most important players in the final establishment of pro- and anti-inflammatory nature of the immune system to set up highly effective host response against pathogenic invaders. Under normal conditions, macrophages usually demonstrate an anti-inflammatory phenotype, with main function of tissue repair [29, 30]. In chronic disorders, such as cancer, macrophages are particularly abundant and are present at all stages of disease progression [31, 32]. Tumor-derived macrophages may play a dual role in cancer; they can either promote tumor growth and metastasis by suppressing immunity and promoting angiogenesis or endorse proimmune and tumoricidal processes [33]. Macrophages at early stages of tumor initiation show M1 phenotype with potential to exhibit antitumor activity, while macrophages in established tumors show M2 phenotype [34]. The M1 phenotype is characterized by the expression of high levels of proinflammatory cytokines, such as TNF-*α*, IL-1*β*, IL-23p19, and IL-6, whereas M2 macrophages demonstrate low IL-12 expression/high IL-10 expression [34–36]. Previously, we demonstrated increase of IL-6, МСР-1, and TNF-*α* cytokine level in murine peripheral macrophages under binase exposure and the absence of elevation of anti-inflammatory cytokine IL-10 level [18]. Here, THP-1-derived macrophages were discovered to be switched to the M1 phenotype under exposure to binase. This resulted in increased expression of TNF-*α* and IL-6, with IL-12 and iNOS inflammatory markers, whereas anti-inflammatory genes Ym1/2 and CD206 were remarkably downregulated (Figure 7()). These results are compatible with cytokine profile of M1, but not M2, macrophages. Thus, one can consider that binase activates macrophages of M1-biased phenotype with tumorocidal properties and stimulates antitumor immunity.

NF-*κ*B activation is required by classical M1 as well as by M2 macrophages differentiation. Regulation of inflammatory responses mainly depends on this transcriptional factor [37]. Here, we observed upregulation of phosphorylation on serine 536 p65 subunit of NF-*κ*B factor after stimulation of macrophages by only binase and RNase1, but not by RNA hydrolysis products (Figure 3()). Sasaki et al. proposed that pp65 is present in the cytoplasm of unstimulated cells, and upon activation, this pp65 translocates to the nucleus with kinetics similar to total p65 [38]. In our study, total p65 expression remained unaltered neither after stimulation of macrophages by binase and RNase1 nor after stimulation by the extracellular RNA and oligonucleotides of different profiles obtained by RNA cleavage by binase or RNase1 (Figures 1(), 3(), 5(), 6()). These data suggested that catalytic activity of RNases do not play significant role in the promotion of NF-*κ*B signaling involving phosphorylation of p65 and potentiation of its transcription activity in macrophages. Although NF-*κ*B activation is a hallmark of inflammatory responses that is frequently detected in tumors [39], some studies indicate that activation of NF-*κ*B in macrophages leads to a reduction in metastases [40] suggesting plasticity in NF-*κ*B functions. Moreover, suppression of the NF-*κ*B activation in in vitro matured human macrophages led to cell death [41]. Taken together, these facts allow us to consider the binase-induced activation of canonical NF-*κ*B pathway in macrophages as beneficial component of antitumor binase effects.

Next, we assessed the effects of RNases, RNA, and RNA fragments on MyD88 adaptor protein expression. MyD88 is a universal adapter protein used by almost all TLRs, except TLR-3, to link members of the TLR and interleukin-1 receptor superfamily for downstream activation of NF-*κ*B and mitogen-activated protein kinases [42]. Upon stimulation with extracellular RNA or RNA fragments, no alterations of macrophages viability and changes in MyD88 expression (Figures 5(a)–5(d)) were found, suggesting that extracellular RNA is not involved in TLR signaling by the MyD88-dependent pathway. This data corresponds well with our previous results demonstrating that extracellular RNA functions as an inducer of inflammatory cytokine response in macrophages independent of TLR-2 and TLR-4 activation [43, 44]. Likewise, no alterations in the MyD88 expression were observed after binase and RNase1 treatment indicating that TLRs which interact with MyD88 did not contribute to RNase-mediated induction of canonical NF-*κ*B pathway.

Additional MyD88-independent modes of regulation for TLR pathways require induction of a number of cytoplasmic adaptor proteins containing TIR domains, including TRIF. TRIF (or TICAM-1) is a TIR-domain containing adaptor protein which activates NF-*κ*B and IRF3 triggering IFN-*β* production; TRIF facilitates signaling through TLR3 and promotes subsequent transcription factors [45]. Following stimulation of macrophages with extracellular RNA and RNases, we found that TRIF adaptor protein did not appear to change its expression at 3 h time point. Thus, extracellular RNA, as well as binase, RNase1, and short oligonucleotides did not seem to initiate signaling events by interacting with TLR 3 pathway.

In addition to well-defined canonical pathway, other mechanisms exist to mediate activation of more specific NF-*κ*B members. We cannot exclude that alternative activation of noncanonical NF-*κ*B pathway could occur in stimulated macrophages. To verify this proposal, additional experiments aimed to detect some components of noncanonical NF-*κ*B pathway, in particular p100, precursor of p52, and active p52 protein, have been performed. Alternative noncanonical NF-*κ*B pathway activates the RelB/p52 NF-*κ*B complex by the means of inducible processing of p100 to p52 [46]. Although two NF-*κ*B pathways were believed to be independent, recent studies uncovered that synthesis of the noncanonical pathway components is controlled by the components of canonical signaling pathway (IKK2-I*κ*B-RelA:p50) [21]. Here, we identified that p100 was expressed at very low level in macrophages treated with extracellular RNA, RNases, and short RNA fragments exclusively after short-term stimulation by binase (Figure 5(e)). The effect was worn off after 1 h of treatment. As no expression of p52 protein, active component of noncanonical NF-*κ*B signaling, was observed, we suggest that neither RNA and RNA fragments nor RNases stimulate noncanonical NF-*κ*B activation. These results suggest the absence of noncanonical NF-*κ*B signaling pathway activation together with canonical NF-*κ*B signaling as beneficial side of RNases therapeutical properties since simultaneous presence of both activated forms of NF-*κ*B serves as an essential mechanism in virus-induced tumorigenesis [47].

## 5. Conclusions

In conclusion, we performed the first attempt to elucidate an effect of exogenous RNase binase on macrophages. We have shown that neither extracellular RNA nor exogenous binase and RNase1 decreased macrophages viability. In this study, we revealed increased expression of activated NF-*κ*B p65 subunit in macrophages upon stimulation by binase and RNase1, but not RNA or short oligonucleotides. Since no changes in MyD88 and TRIF adaptor protein expression were observed, it might have been assumed that TLRs did not contribute in NF-*κ*B pathway activation by binase and RNase1. However, this assumption needs to be confirmed in further studies. Moreover, we have shown that binase acts as a proinflammatory agonist mediating antitumor macrophage response by inducing M-1 macrophage polarization.
